# Prediction of protein-binding areas by small-world residue networks and application to docking

**DOI:** 10.1186/1471-2105-12-378

**Published:** 2011-09-26

**Authors:** Carles Pons, Fabian Glaser, Juan Fernandez-Recio

**Affiliations:** 1 Joint BSC-IRB research programme in Computational Biology, Barcelona Supercomputing Center, Barcelona 08034, Spain; 2 Computational Bioinformatics, National Institute of Bioinformatics (INB), Barcelona 08034, Spain; 3 Bioinformatics Knowledge Unit, The Lorry I. Lokey Interdisciplinary Center for Life Sciences and Engineering, Technion, Haifa 32000, Israel

**Keywords:** protein interactions, small-world networks, binding site prediction, protein-protein docking, pyDock

## Abstract

**Background:**

Protein-protein interactions are involved in most cellular processes, and their detailed physico-chemical and structural characterization is needed in order to understand their function at the molecular level. In-silico docking tools can complement experimental techniques, providing three-dimensional structural models of such interactions at atomic resolution. In several recent studies, protein structures have been modeled as networks (or graphs), where the nodes represent residues and the connecting edges their interactions. From such networks, it is possible to calculate different topology-based values for each of the nodes, and to identify protein regions with high centrality scores, which are known to positively correlate with key functional residues, hot spots, and protein-protein interfaces.

**Results:**

Here we show that this correlation can be efficiently used for the scoring of rigid-body docking poses. When integrated into the pyDock energy-based docking method, the new combined scoring function significantly improved the results of the individual components as shown on a standard docking benchmark. This improvement was particularly remarkable for specific protein complexes, depending on the shape, size, type, or flexibility of the proteins involved.

**Conclusions:**

The network-based representation of protein structures can be used to identify protein-protein binding regions and to efficiently score docking poses, complementing energy-based approaches.

## Background

Protein-protein interactions are fundamental to many cellular processes [1], and a detailed atomic-level description of protein complexes would be needed in order to fully understand their association mechanism [2]. The inherent technical difficulties of experimental methods to solve the three-dimensional structure of many protein complexes [3] call for the integration of complementary computational approaches [4,5]. However, the structural prediction of the complex formed by two interacting proteins remains one of the most challenging problems in computational biology. The complex nature of the rotational, translational, and conformational search makes this task extremely difficult and too costly in computational terms to be addressed purely by full-atom molecular mechanics simulations. Therefore, different simplifications are required in order to approach the docking problem [6]. The treatment of proteins as rigid bodies or their description at low resolution (either in grids [7-10] or coarse-grained models [11-13]) are common simplifications in almost all docking approaches, at least in their initial stages. Additionally, the efficient combination of different scoring terms can increase the overall quality of the predictions if they identify different contributions to binding [13]. Therefore, a way to improve the performance of current scoring functions is the detection of new descriptors for protein binding, orthogonal to existing ones, which could be easily integrated in the scoring phase.

Recently, the analysis of protein structures as small-world network systems has attracted significant interest [14-17]. In small-world networks (i) the average shortest path (between any two nodes) is logarithmically related to the total number of nodes, and (ii) a large average clustering coefficient is observed [18]. Using this approach, proteins can be modeled as a network of interactions, where the nodes represent residues and the edges their contacts. It is assumed that highly connected regions of the network play a key role in the protein, which can be easily detected by means of different topology-based network parameters. Indeed, topological data based on small-world network descriptions of proteins have been recently exploited to predict protein-protein interfaces [19,20], protein-DNA interfaces [21], protein-RNA interfaces [22], ligand binding sites [23,24], modeling [25], protein dynamics [26], protein disorder [27], ribosome functional sites [28], to identify critical residues for protein function [15], or to evaluate protein docking poses [29].

In this work, we characterized unbound proteins as small-world networks for their use in docking. We used different topology measures and evaluated their use to predict protein binding sites. We then applied these descriptors to the scoring stage of protein-protein docking using the latest standard docking benchmark. These scoring functions were integrated in pyDock, a successful docking scoring algorithm based on physico-chemical terms [30].

## Results and discussion

### Interface prediction by network-based parameters

We modeled each of the unbound protein structures of the docking benchmark 3.0 [31] as residue-based networks based on Cα atoms. We then calculated different topology-based parameters for all nodes of the network and mapped their values into the residues they represented (see Methods). For comparison purposes, we also generated topology networks based on the Cβ atoms. The *closeness *and *degree *values were virtually the same for the two types of networks (correlation *r*^2 ^= 0.97 and 0.92, respectively), with some differences in the *clustering *and *betweenness *parameters (correlation *r*^2 ^= 0.58 and 0.49, respectively). In the next section we describe how we directly used these values for docking scoring, with no further optimization. But first, we have evaluated the capabilities of the network-based values to predict binding interfaces. With this only purpose, for each protein and network parameter, we defined as interface predictions an arbitrary number of residues (i.e. nodes) with the highest network topology values (see below) and evaluated whether they were present in the binding site of the known protein complex. For this purpose of interface predictions, only surface residues of the unbound protein were considered, defined as those having relative accessible surface area (ASA) > 0.1%. The positive predictive value (PPV) for each complex was calculated as the percentage of predicted residues that were part of the real interface (i.e. residues with at least one atom within 10 Å of the partner protein in the complex). Then we computed the mean PPV of all unbound proteins. Additionally, we used different cutoff values to restrain the selection of predicted residues. It should be noted that some proteins had no residues with values above certain cutoffs and, thus, no predictions were computed in these cases. The random PPV was calculated by randomly selecting surface residues of the unbound proteins. This was repeated 100 times for the different cutoff values.

**Figure 1:**
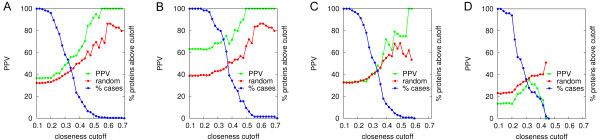
**Binding site prediction with *closeness***. Positive predicted value (PPV) of binding site predictions based on *closeness *parameter, considering only the residues with the top four *closeness *scores that were above the cutoff value indicated in abscissas. Random PPV is shown for comparison. The percentage of proteins that have any residue with a *closeness *value above the cutoff is shown ("% cases"). Data calculated for (A) all proteins in benchmark 3.0; (B) only enzyme/inhibitor cases (20.2% of the benchmark); (C) only "other" cases (51.6% of the benchmark); (D) only antibody/antigen cases (28.2% of the benchmark).

We first studied the results of the interface predictions based on the arbitrary number of four residues with the highest network *closeness *parameter at different cutoff values (see Figure 1A). Results did not significantly change when considering the residues with the top one, two, and ten *closeness *values (see additional file 1: Figure S1). The higher the cutoff *closeness *values, the better the predictions, achieving 48.8%, 62.0% and 91.7% PPV at 0.30, 0.40 and 0.50 cutoff *closeness *values, respectively. In parallel, the percentage of the total proteins that showed predictions decreased to 58.1%, 19.4% and 3.6%, respectively. However, random PPV also improved with the cutoff. This behavior is a consequence of the *closeness *definition (see Methods). The average distance to all other nodes is expected to be always higher in larger proteins than in smaller ones. Thus, the higher *closeness *values were mostly found in the smallest proteins (see additional file 1: Figure S2A), in which it was easier to select by chance an interface residue (defined above). Indeed, proteins that do not contain any residue with *closeness *value above a given arbitrary threshold (e.g. 0.2) were all large (i.e. more than 400 residues) and in some cases presented domains weakly connected with the protein core, like the receptor of the FH2 complex (PDB code 1Y64). By residue type, the largest average *closeness *values typically correspond to hydrophobic residues (e.g. CYS, ILE, VAL, PHE, TYR, or LEU), probably due to their higher frequency in the protein interfaces, as well as in the protein core. In any case, the difference of the predictive success rates with respect to random was significant and increased with the cutoff values. The most successful predictions were found for the enzyme/inhibitor group, with a mean PPV of 63.3% when considering all proteins, and reaching 100% when applying cutoff values over 0.50 to the *closeness *values. In these cases, success rates were always clearly above random (see Figure 1B). The proteins classified in the benchmark as "other" had PPV similar to the expected by random distribution, although the success rates were slightly better than random at higher cutoff values (see Figure 1C). On the other hand, interface predictions for antibodies and antigens were always worse than the expected random PPV, and more surprisingly, success rates even decreased at higher cutoff values (see Figure 1D). Actually, in most antibodies the higher *closeness *values were found in the concave surface formed by the two antibody chains instead of in the CDR, which completely misled our predictions. The success of the predictions for the antigens showed a similar trend to the "other" group of proteins, as expected, given that antigens have not evolved to bind antibodies. The interface predictions with the networks generated from the Cβ atoms were virtually the same (average PPVs were only around 5% worse than those from Cα based networks; data not shown). This shows that predictions are not very sensitive to whether the networks are defined from the Cα or Cβ atoms, and for the rest of the analysis we will only use the networks defined with the Cα atoms.

**Figure 2:**
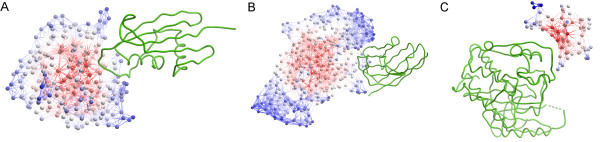
**Examples of protein binding site predictions**. Network representation of three proteins shown as balls (nodes) and sticks (edges connecting nodes). Nodes are colored by their *closeness *value (from minimum values in blue to maximum values in red). All partner proteins are shown in green ribbon. (A) A successful prediction for the receptor of the enzyme/inhibitor complex between Substilisin and Streptomyces subtilisin inhibitor (PDB code 2SIC). The top four residues according to the *closeness *parameter are part of the interface where the ligand binds. (B) Prediction for the receptor of antibody/antigen between the camel VHH and Pancreatic alpha-amylase (PDB code 1KXQ). The prediction is unsuccessful because residues with high *closeness *values are in the protein core, far from the binding site. (C) Ligand of the "other" type complex between CDK2 kinase and the human cyclin dependent kinase subunit (PDB code 1BUH), in which all four predicted residues are in the binding site.

We have also computed the interface prediction success rates for three additional network-based parameters (see additional file 1: Figures S3, S4 and S5). The predictive results based on the *degree *network parameter showed a similar trend to those of *closeness*, where PPV improved at higher (i.e. more restrictive) cutoff values. The *degree *of a given node is the fraction of nodes to which such node is connected, so residues in small proteins will expectedly have higher *degree *values (see additional file 1: Figure S2B). Thus, residues selected at higher cutoff values come mostly from small proteins, in which random PPV is expected to be higher, as it happened with *closeness*. On the other hand, results with the *clustering *parameter worsened as the cutoff increased. High *clustering *values were mostly present in bigger proteins (see additional file 1: Figure S2C), in which it was more difficult to detect the correct binding site by chance. Values for *betweenness *tended to be lower for smaller proteins, but they were much less dependent on size than the rest of parameters (see additional file 1: Figure S2D). Thus, interface predictive success rates based on *betweenness *were less determined by the cutoff applied. All topological parameters yielded better results than average for the enzyme/inhibitor group, and worse for the antibody/antigen cases. Only in the case of *degree*, the PPV for the antibody/antigen group was above random, since the rather local *degree *values were not concentrated in the concave surface formed by the two antibody chains, as opposed to what happened with the rest of network-based parameters. Examples of predictions for each complex type are shown in Figure 2, with residues colored by their *closeness *value.

### Network-based scoring of docking poses

**Figure 3:**
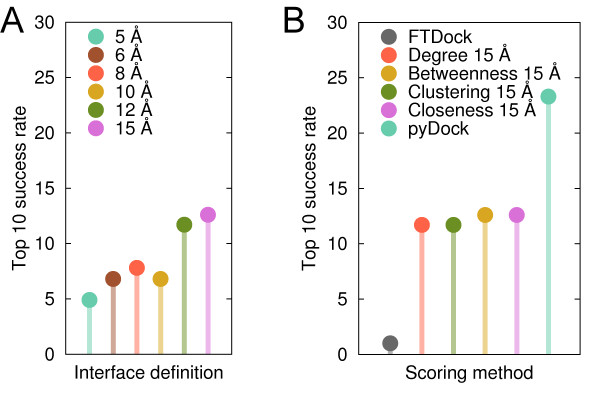
**Performance of network-based scoring methods**. (A) Top 10 success rates when scoring the docking poses with the *closeness *values using different contact distances to define the docking interface residues. (B) Best top 10 success rates achieved by the different network-based parameters. Results by FTDock and pyDock are shown for comparison purposes.

Having tested the capabilities of residue-based network parameters to predict interface residues, we further explored their application to score rigid-body docking poses generated for the docking benchmark 3.0. Figure 3A shows the top 10 success rates (i.e. percentage of cases with a near-native solution within the 10 best-scoring docking poses) obtained by scoring docking poses with the *closeness *values of the docking interface residues only. These interface residues (including surface and buried ones) were defined at different contact distances between all heavy atoms of the complex subunits (see Methods). Success rates improved when large docking interfaces were considered, reaching 12.6% when including residues up to 15 Å from the docking partner. However, using even larger docking interfaces worsened the top 10 success rate (e.g. 10.7% using 18 Å). Scoring with other network-based parameters showed similar trends (see additional file 1: Figure S6), where the best results were always obtained with docking interfaces defined at 15 Å (see Figure 3B). The top 10 success rates were 12.6% for *Betweenness 15 Å*, and 11.7% for *Clustering 15 Å *and *Degree 15 Å*.

Additionally, we evaluated how the docking scoring performance depended on the success of the interface predictions for each partner. Notably, the 29 cases (out of 124) for which *closeness *correctly predicted the interface in both partners achieved a top 10 docking success rate of 34.6%, clearly above average. On the other hand, in the 46 cases in which only one of the partners had a correct interface prediction, the docking predictions were of worse quality (top 10 success rate 7.7%). Finally, when both partners had incorrect interface predictions (49 cases), docking success clearly worsened (2.6%). The correlation between the success of the interface predictions and the docking scoring performance is thus evident.

### Combined energy-based and network-based scoring

Scoring with topology-based network parameters was in all cases clearly better than the FTDock default scoring (top 10 success rate 1%), but still far from the performance achieved by state-of-the-art energy-based scoring functions like pyDock (see Figure 3B). Interestingly, the correlation between the results obtained by these two different types of scoring functions (physics-based pyDock and topology-based network) was very low (below 0.15 for all network parameters), which suggests that they are detecting different contributions to binding.

**Figure 4:**
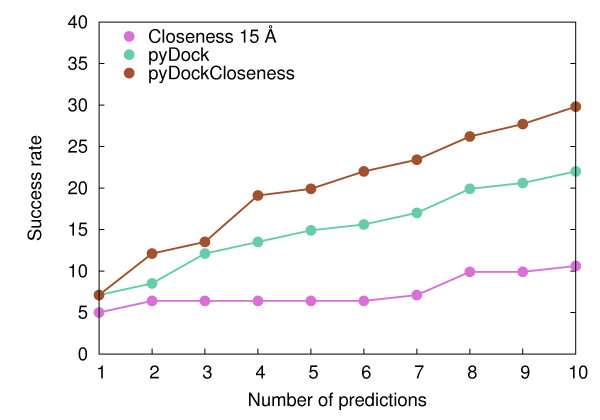
**Performance of the combination of energy-based and network-based scoring methods**. Success rates for the top 1 to 10 predictions of *Closeness 15 Å*, pyDock and their combined score pyDockCloseness.

Taking this into account, we combined pyDock and the best conditions found for the four network-based scoring functions (using contact distance 15 Å; see Figure 3B) by weighting the values of the network-based contribution (see Methods). All combinations of network-based scoring and pyDock gave better top 10 success rates than pyDock alone (see additional file 1: Figure S7). The best results were obtained when combining *Closeness 15 Å *and pyDock, reaching a top 10 success rate of 31.1%, substantially better than pyDock alone (23.3%). This combined scheme was implemented in a new scoring function called pyDockCloseness. We performed a cross-validation test to discard any possibility of over-training in our scoring function (see Methods). In all cases the resulting optimal weight was the same (*w *= 0.45), which confirmed the robustness of the combined function. As a further test to prevent over-fitting, we validated our combined scoring function on the new cases of the recently released docking benchmark 4.0 [32]. FTDock found a near-native solution in 38 out of these 52 new cases. Top 10 success rate was 18.4% for pyDock, 5.3% for *Closeness 15 Å *and 26.3% for pyDockCloseness, confirming the improvement of the combined scored achieved in benchmark 3.0. Taking into account all the cases in benchmark 4.0 (which encompasses the whole benchmark 3.0, and that based on the above cross-validation test, can be safely used for the rest of the analysis in this work), FTDock found a near-native solution in 141 out of 176 cases and top 10 success rates were 22.0%, 10.6% and 29.8% for pyDock, *Closeness 15 Å *and pyDockCloseness, respectively (see Figure 4). This represents a 36% improvement of pyDockCloseness with respect to pyDock. In a recent study [29] RosettaDock results were combined with a network-based scoring, achieving an improvement of 15% with respect to RosettaDock alone for 43 docking cases (a sub-set of the benchmark used here, for which our results are similar to those for the whole set in the present study). In that work, two different amino acid networks were generated for every single docking pose, as opposed to our method, in which we pre-compute residue-based network parameters just once for the unbound subunits.

### Analysis by complex type

**Figure 5:**
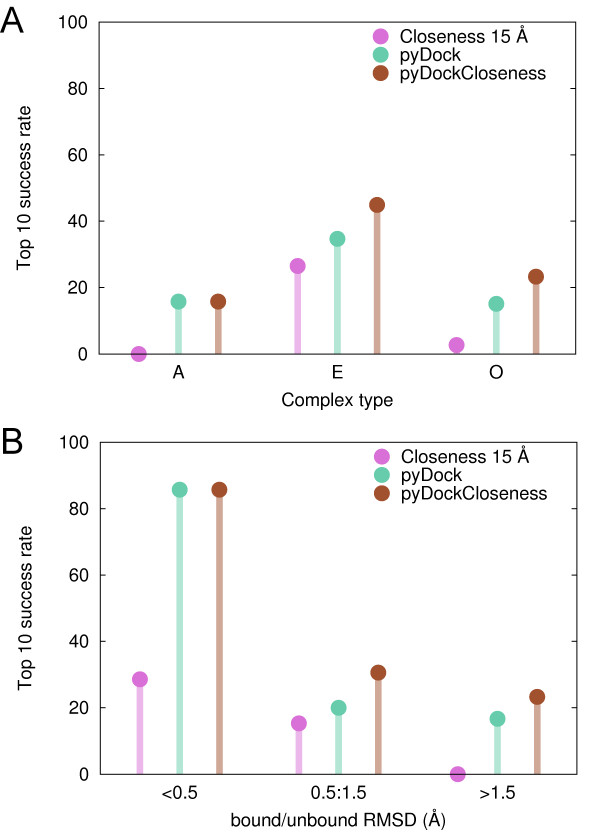
**Scoring performance of pyDock, *Closeness 15 Å *and pyDockCloseness by complex type and conformational change upon binding**. Top 10 success rates by (A) complex type, (B) averaged bound/unbound RMSD of receptor and ligand.

The ability of *closeness *to identify interface residues strongly depended on the type of complex (see Figure 1). As expected, the same trend was observed in the success rates of pyDockCloseness for the scoring of docking poses (see Figure 5A; based on benchmark 4.0, as in the rest of the work). *Closeness 15 Å *showed poor results in the scoring of the antibody/antigen cases, but the combined score did not worsen pyDock results (15.8% top 10 success rate). The scoring results on the enzyme/inhibitor group substantially improved with all parameters. For example, *Closeness 15 Å *had a remarkable 26.5% top 10 success rate, not far from that of pyDock (34.7%). Moreover, the combination of both scores clearly increased the top 10 success rate (44.9%). In the case of the complexes classified as "other", *Closeness 15 Å *performed poorly (2.7% top 10 success rate) as compared to pyDock (15.1%). However, their combination significantly improved the success rate to 23.3%. Given the poor interface predictions and docking results of the network-based parameters in antibody/antigen cases, we repeated the weight optimization between *Closeness 15 Å *and pyDock considering enzyme/inhibitors and "other" type of complexes only. The resulting weighting factor was the same as with the whole set of complexes, reinforcing the robustness of our pyDockClosenesss scoring function. In any case, antibody/antigen cases were discarded for the rest of the analyses.

### Coarse-grained model and conformational changes upon binding

The improvement of pyDockCloseness over pyDock was noteworthy in cases with significant conformational change (see Figure 5B). We have previously reported the strong dependency of pyDock success rates on the flexibility of proteins [33]. Indeed, top 10 success rate was excellent (85.7%) for cases with small conformational changes, but then it substantially dropped for the rest of cases. *Closeness 15 Å *behaved similarly, yielding top 10 success rates of 28.6%, 15.3% and 0% for the groups of proteins with small, medium and large conformational changes upon binding, respectively. In the cases with small changes upon binding (averaged unbound/bound RMSD for receptor and ligand < 0.5 Å), *Closeness 15 Å *contribution to the combined score could not improve the already excellent results of pyDock. On the contrary, in cases showing medium conformational changes (unbound/bound RMSD between 0.5 and 1.5 Å), pyDockCloseness top 10 success rate (30.6%) was considerably better than that of pyDock alone (20.0%). Interestingly, for the most difficult and challenging group of cases with high flexibility (unbound/bound RMSD >1.5 Å), the *Closeness 15 Å *contribution to the combined score made the success rate to improve with respect to pyDock (from 16.7% to 23.3%), regardless of the poor performance of the network-based scoring alone for the top 10 predictions (0%). Altogether, our coarse-grained network-based approach (only Cα atoms were used to build the networks and the scoring was at residue level, see Methods) seems to be especially helpful in cases with significant conformational changes, successfully complementing our all-atom approach whose predictions quickly degenerated in inaccurate geometries [33].

### Size and anisotropy

The size and shape of a given protein determine the general topology of the network-based representation, and in consequence, the parameters derived from that are expected to show different features. Therefore, it was of interest to analyze how the different scoring schemes were affected by the size and anisotropy of the proteins.

**Figure 6:**
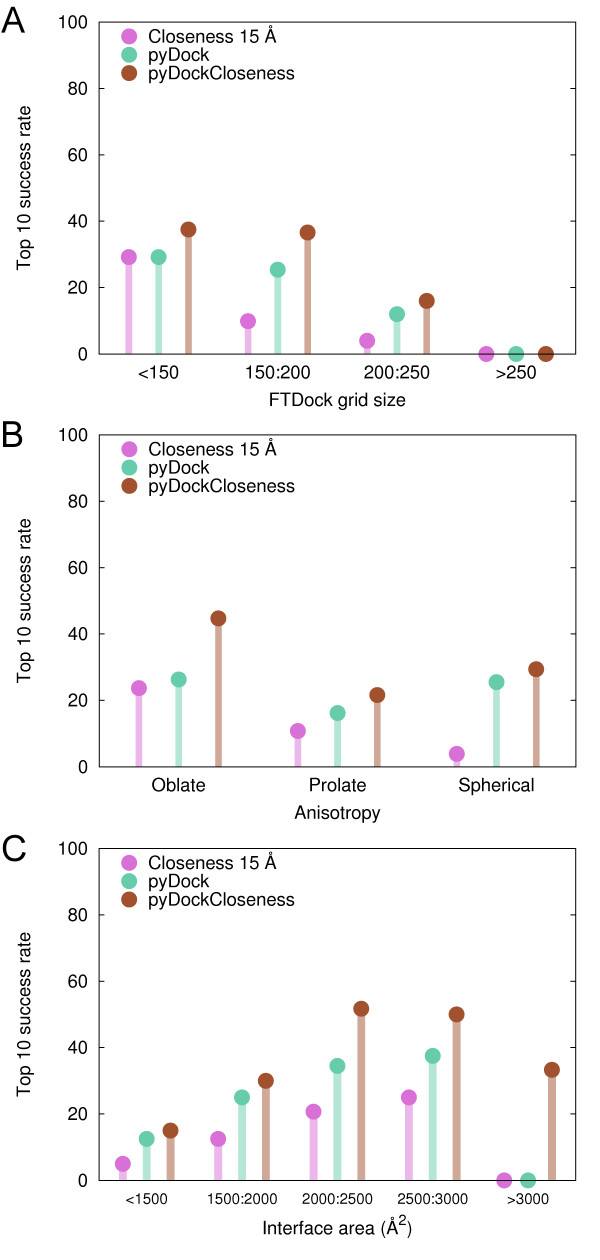
**Scoring performance of pyDock, *Closeness 15 Å *and pyDockCloseness by size, shape and interface**. Top 10 success rates according to (A) FTDock grid size (proportional to the sum of both protein radius), (B) anisotropy of the proteins, (C) interface size of the complexes.

Cases in the benchmark were classified according to the FTDock grid size, which is proportional to the sum of both protein radii [7]. The top 10 success rate obtained by *Closeness 15 Å *matched that of pyDock for smaller proteins (29.2% for cases with grid size < 150; see Figure 6A), a remarkable result explained by the fact that residues with high *closeness *values were close to the surface of the protein and made a more specific contribution when scoring docking candidates. Bigger proteins tend to have the higher *closeness *values more buried, making their contribution to the selection of docking poses more indefinite. Indeed, as the size increased the performance of *Closeness 15 Å *worsened faster than pyDock. The poor success rates in the group of the largest proteins (grid size >250) was due to the limited sampling of FTDock in these conditions [33]. For proteins with grid size < 250, pyDockCloseness was better than either pyDock or *Closeness 15 Å *individual scorings, reaching 37.5%, 36.6% and 16% top 10 success rate for the small, medium and large grid-size groups, respectively.

The anisotropy of the proteins (i.e. the length of the most different axis divided by the mean length of the other two) played a crucial role in the success of the *Closeness 15 Å *scoring. Spherical cases (those where receptor and ligand had anisotropy values between 0.7 and 2.0) showed a poor performance, whereas prolate cases (those where either receptor or ligand had anisotropy value above 2.0) and, specially, oblate cases (those where either receptor or ligand had anisotropy value below 0.7) yielded better predictions (top 10 success rates were 3.9%, 10.8% and 23.7% for spherical, prolate and oblate cases, respectively; see Figure 6B). In spherical proteins, high *closeness *values tended to be in the core of the protein, which made difficult for these residue values to contribute to the scoring of near-native poses in a specific manner. Interestingly, this suggests that non-spherical proteins have general topological features that are recognized by the partner. This seems to be in contradiction with a recent work in which a new local closeness measure was defined in order to overcome the lack of predictive ability of global closeness (the measure that we use in this study) for protein-ligand binding sites in non-globular proteins [34]. On the contrary, our results for anisotropic proteins clearly outperformed those obtained for spherical proteins. This perhaps reflects the different nature of the protein-protein and protein-ligand binding problem. On the other hand, pyDock performance was less affected by anisotropy (25.5%, 16.2% and 26.3% for spherical, prolate and oblate cases, respectively). In this case, the worse results of the prolate cases were probably due to the poorer sampling of FTDock (prolate cases tended to be larger than average in the benchmark). Nevertheless, success rates for pyDockCloseness improved those of the individual scorings, reaching top 10 success rates of 29.4%, 21.6% and 44.7% respectively.

### Performance by interface area

We also found a strong correlation between the interface size of the complexes and the top 10 success rates of the scoring methods (see Figure 6C). Cases with very small or very large interfaces showed the worse predictions. Top 10 success rates steadily increased with the interface size for pyDock and *Closeness 15 Å*, but dramatically dropped for the group of largest interfaces (0% with pyDock and *Closeness 15 Å*. Notably, for this group pyDockCloseness showed top 10 success rate of 33.3%, emphasizing the complementarity effect of both individual scoring functions. It is also interesting that topological network parameters can give such predictive trends, similar to energy-based functions.

## Conclusions

In this work, we have shown that network topology values can be used to identify binding regions in proteins. Predictions were significantly better than random in all complex types except in the antibody/antigen cases, where the highest *closeness *values were generally found in the concave surface formed by the two antibody chains. We have also analyzed in detail the potential use of such network topology parameters as scoring functions to identify near-native docking poses according to different interface definitions. Good performance was achieved for small, oblate and enzyme proteins, similar to that of physical-based methods like pyDock. However, the results from both types of scoring functions were found to be complementary and synergistic. Thus, the combination of the network-based scoring *Closeness 15 Å *and pyDock improved the latter top 10 success rate by 36% as tested in the most updated standard benchmark. This improvement was much larger for oblate proteins, complexes with large interfaces and cases classified as "other", in which energy-based pyDock typically had the worst results. More importantly, the coarse-grained representation in the network-based scoring made it possible to improve the predictive success in the most challenging type of docking cases, that is, those with significant conformational changes upon binding. Although this approach has limitations in cases with certain topological features, like spherical or very large proteins, we have shown here its potential applications for docking as a complement to energy-based approaches.

## Methods

### Representation of proteins as residue networks

In this work, unbound proteins were modeled as topological networks as follows. The nodes represented the Cα atoms of all the residues in a protein, and the edges the residues in contact (i.e. those whose Cα atoms were within 8.5 Å distance [35]). To construct the graph topology and calculate the four centrality parameters analyzed in this work, we used the NetworkX python package [36]. For comparison purposes, we also generated topological networks based on the Cβ atoms instead (Cα for Glycine). The resulting networks were very similar (see additional file 1: Figure S8) and the predictions from these networks were virtually the same (see Results).

### Graph Theory

Within graph theory and network analysis, there are various node measures that determine its importance. In this work, we tested four widely used network parameters: three centrality measures (*betweenness*, *closeness *and *degree*) and the *clustering *coefficient. The *closeness *centrality of a node *x *is defined as follows:

(1) closeness(x)=(N−1)/∑d(x,y)

where *N *is the total number of nodes in the network and *d(x, y) *is the shortest path distance between node *x *and any other node *y*. Thus, the *closeness *of node *x *is the inverse of the average distance to all other nodes. The three remaining network parameters are defined as follows: for any node *x*, *degree *is the number of edges incident to that node, *betweenness *is the sum of the fraction of all shortest paths between any two nodes that pass through *x *and *clustering *is the fraction of contacts that exist between its neighbors (i.e. the number of triangles through *x*) relative to the maximum possible contacts between them. For this work we showed the inverted *clustering *value (1/*clustering*) so that higher scores correlate with protein binding sites.

### Benchmark sets

We used the standard protein-protein docking benchmark 3.0 [31] for (i) the assessment of the use of network-based parameters for binding site prediction, (ii) the comparison of the different topological parameters for docking scoring, and (iii) the training of the optimal balance between pyDock and the network-based scoring. The new cases in benchmark 4.0 (the latest so far) [32] were used to validate the optimal balance found between pyDock and the network-based scoring. Benchmark 4.0 (which includes the cases of benchmark 3.0) was used for the performance analysis.

### Generation of docking poses

We used FTDock [7] with standard parameters (using electrostatics and 0.7 Å grid resolution) to generate 10,000 rigid-body docking poses for the 176 unbound cases of the latest standard protein-protein docking benchmark [32]. A docking pose was considered a near-native solution if its ligand Cα-RMSD with respect to the crystal structure was below 10 Å. The success rate for the top 10 predictions was calculated as the percentage of cases in the benchmark that had a near-native solution within the first 10 predictions. For this calculation, only the cases for which FTDock generated at least one near-native solution were considered (103 for benchmark 3.0 and 141 for benchmark 4.0).

### Scoring by network parameters

We scored docking poses using the topology-based parameters precalculated on the unbound proteins (see above). Only the values of residues at the docking interface were used. For instance, to obtain the *Closeness *score for a given docking pose *P*, the precomputed *closeness *values of all interface residues *i, j *(defined as those with a heavy atom within a threshold distance *d *from any heavy atom of the partner protein), from receptor and ligand respectively, were added up. Each residue value was added only once, regardless of the number of contacts that formed with the partner protein. Weighting the residue-level *closeness *values by the number of atomic contacts established with the partner protein worsened the scoring results. A possible reason is that we are scoring rigid-body docking poses, in which side-chains are not always in optimal conformation for binding, and therefore a coarse-grained scoring based on counting residues (not atomic contacts) is preferred. Perhaps flexible docking solutions might benefit from a scoring system based on the number of contacts, but this is beyond the scope of the current work. The same scheme was applied to the rest of network parameters. Different values for *d*, ranging from 5 to 15 Å were tested:

(2) $$Closeness_{P}^{d}=\underset{i}{\sum }closeness_{i}+\underset{j}{\sum }closeness_{j}$$

### pyDock

pyDock [30] is a scoring function that evaluates the binding energy of rigid-body docking poses, taking into account the contributions of the desolvation, electrostatics and van der Waals energy terms. The desolvation is ASA-based and uses atomic solvation parameters. Coulombic electrostatics is calculated with a distance-dependent dielectric constant, and individual contributions are truncated to ±1 kcal/mol to avoid artificial high scores from models with overlap proteins. The van der Waals term is based on a 6-12 Lennard-Jones potential, weighted to 0.1. Interatomic potentials are truncated to +1 kcal/mol to avoid excessive penalization for models containing clashes. To calculate the electrostatics and the van der Waals terms AMBER94 parameters are used. This scoring function showed excellent results in several CAPRI rounds [37,38] and in external benchmarks [33].

### Combining pyDock and network-based scoring

We combined each network-based scoring and pyDock in a new scoring function. For instance, for a given docking pose *P *and a threshold distance *d*, we defined the pyDockCloseness score as the combination of *Closeness *and pyDock:

(3) $$\begin{array}{c} pyDockCloseness_{P}^{d}=pyDock_{P}+\\ wCloseness_{P}^{d} \end{array}$$

The value of *w *was calculated by minimizing the function F(w) on benchmark 3.0 [31], a subset of the latest protein docking benchmark (see above):

(4) $$F\left(w\right)=\underset{m}{\sum }\text{ln}\left(Rank_{m}^{w}\right)$$

where $Rank_{m}^{w}$ was defined as the best rank of a near-native solution (ligand RMSD < 10 Å) for the benchmark case *m*, using *w *to balance the *Closeness *scoring in the pyDockCloseness function. Values ranging from 0.0 to 2.0 with a step of 0.05 were used to determine the lowest value of F(w).

In order to prevent overfitting, we validated the predictions on the subset of benchmark 4.0 that was not used for the training of *w*. In addition, we performed a leave-one-out cross-validation to ensure the optimized parameter was robust to permutations. The process consisted in calculating *w *using all the cases of the training set except one, which was then used for validation. This was repeated in a way that each case in the training set was used once for validation.

## Authors' contributions

CP performed the docking calculations. FG calculated the topological parameters of proteins and devised the concept. CP and FG drafted the manuscript. JFR devised the concept, directed the research and finalized the draft. All authors analyzed the results, read and approved the final manuscript.

## Supplementary Material

Additional file 1 **Supporting figures**. this file contains all the supporting figures that are referenced in the text. Click here for file
