# Supplementary material for: Prediction of protein-binding areas by small-world residue networks and application to docking
Source: BMC Bioinformatics. 2011 Sep 26;12:378. doi: 10.1186/1471-2105-12-378 (PMC3189935; doi:10.1186/1471-2105-12-378)
Supplement: Additional file 1 — Supporting figures. this file contains all the supporting figures that are referenced in the text. [file 1471-2105-12-378-S1.DOC]

**
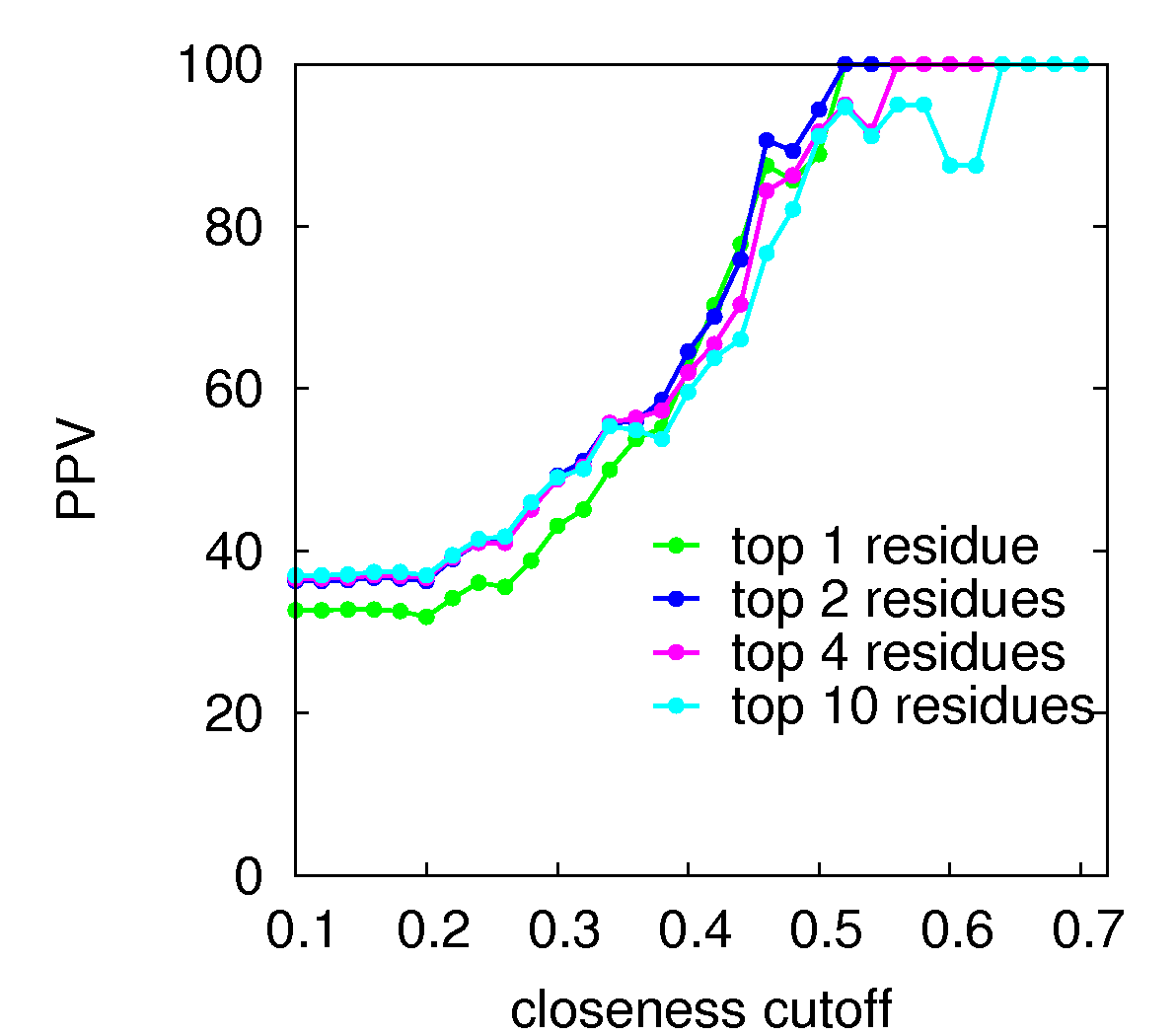
**

**Figure S1.- Binding site prediction with *closeness***

PPV of binding site predictions considering the residues with the top 1, 2, 4, and 10 *closeness* values. Only values above the cutoff indicated by the abscissas are taken into account


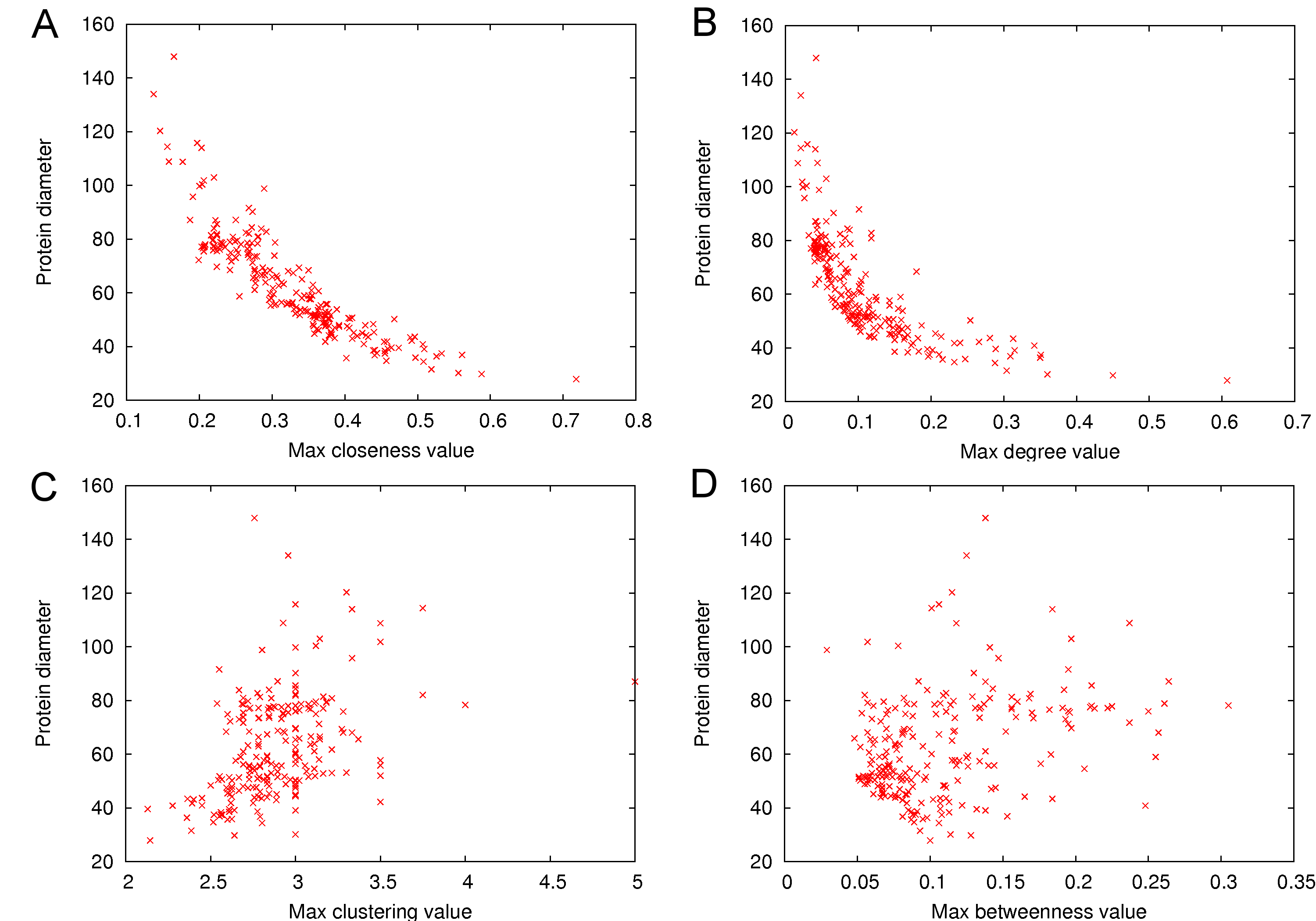


**Figure S2.- Correlation of topological values and protein size**

Protein diameter vs. the maximum value per protein of (A) closeness, (B) degree, (C) clustering and (D) betweenness


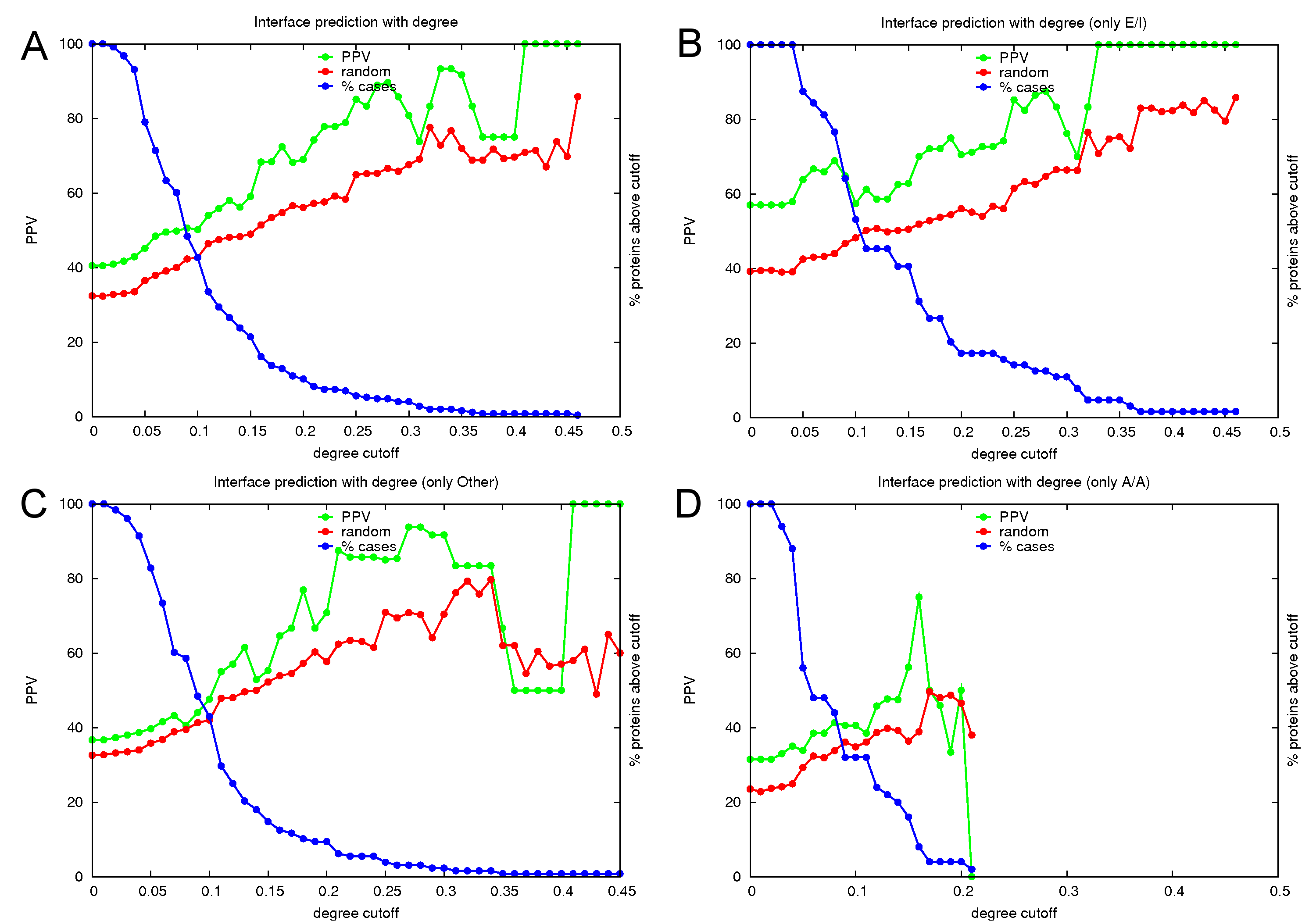


**Figure S3.- Binding site prediction with *degree***

Positive predicted value (PPV) of binding site predictions based on *degree* parameter, considering only the residues with the top four *degree* scores that were above the cutoff value indicated in abscissas. Random PPV is shown for comparison. The percentage of proteins that have any residue with a *degree* value above the cutoff is shown (“% cases”). Data calculated for (A) all proteins in benchmark 3.0; (B) only enzyme/inhibitor cases; (C) only “other” cases; (D) only antibody/antigen cases.


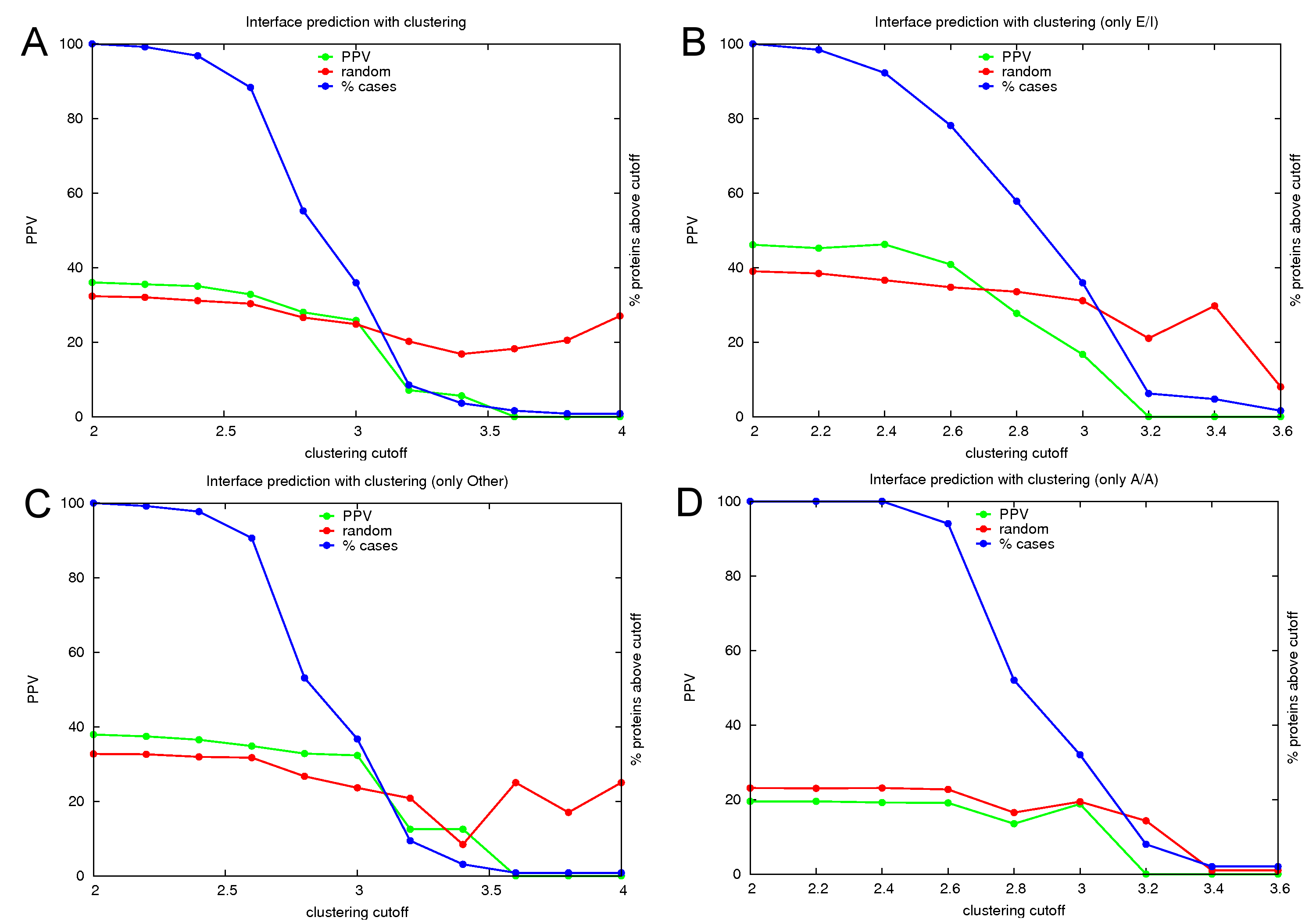


**Figure S4.- Binding site prediction with *clustering***

Positive predicted value (PPV) of binding site predictions based on *clustering* parameter, considering only the residues with the top four *clustering* scores that were above the cutoff value indicated in abscissas. Random PPV is shown for comparison. The percentage of proteins that have any residue with a *clustering* value above the cutoff is shown (“% cases”). Data calculated for (A) all proteins in benchmark 3.0; (B) only enzyme/inhibitor cases; (C) only “other” cases; (D) only antibody/antigen cases.


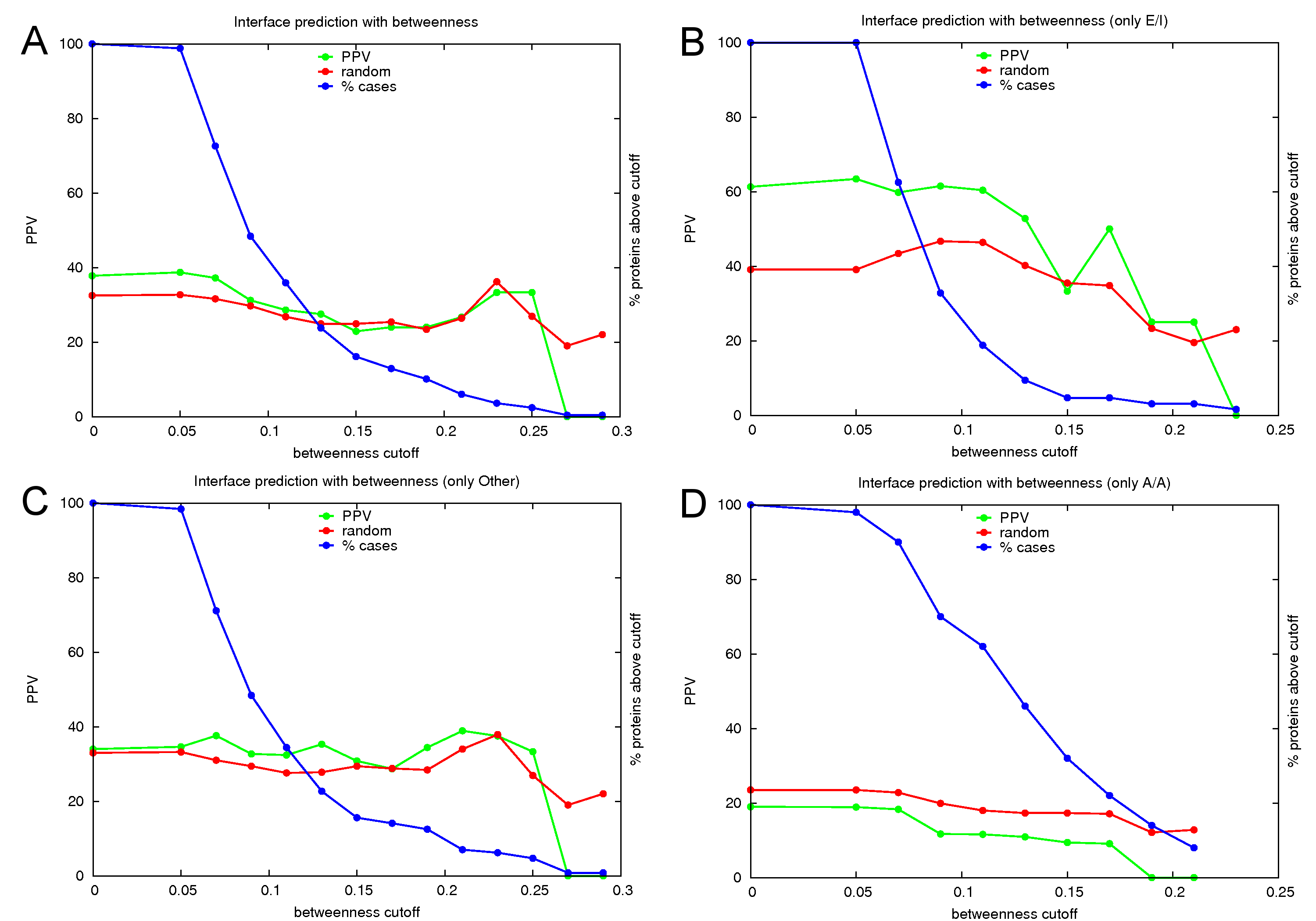


**Figure S5.- Binding site prediction with *betweenness***

Positive predicted value (PPV) of binding site predictions based on *betweenness* parameter, considering only the residues with the top four *betweenness* scores that were above the cutoff value indicated in abscissas. Random PPV is shown for comparison. The percentage of proteins that have any residue with a *betweenness* value above the cutoff is shown (“% cases”). Data calculated for (A) all proteins in benchmark 3.0; (B) only enzyme/inhibitor cases; (C) only “other” cases; (D) only antibody/antigen cases.


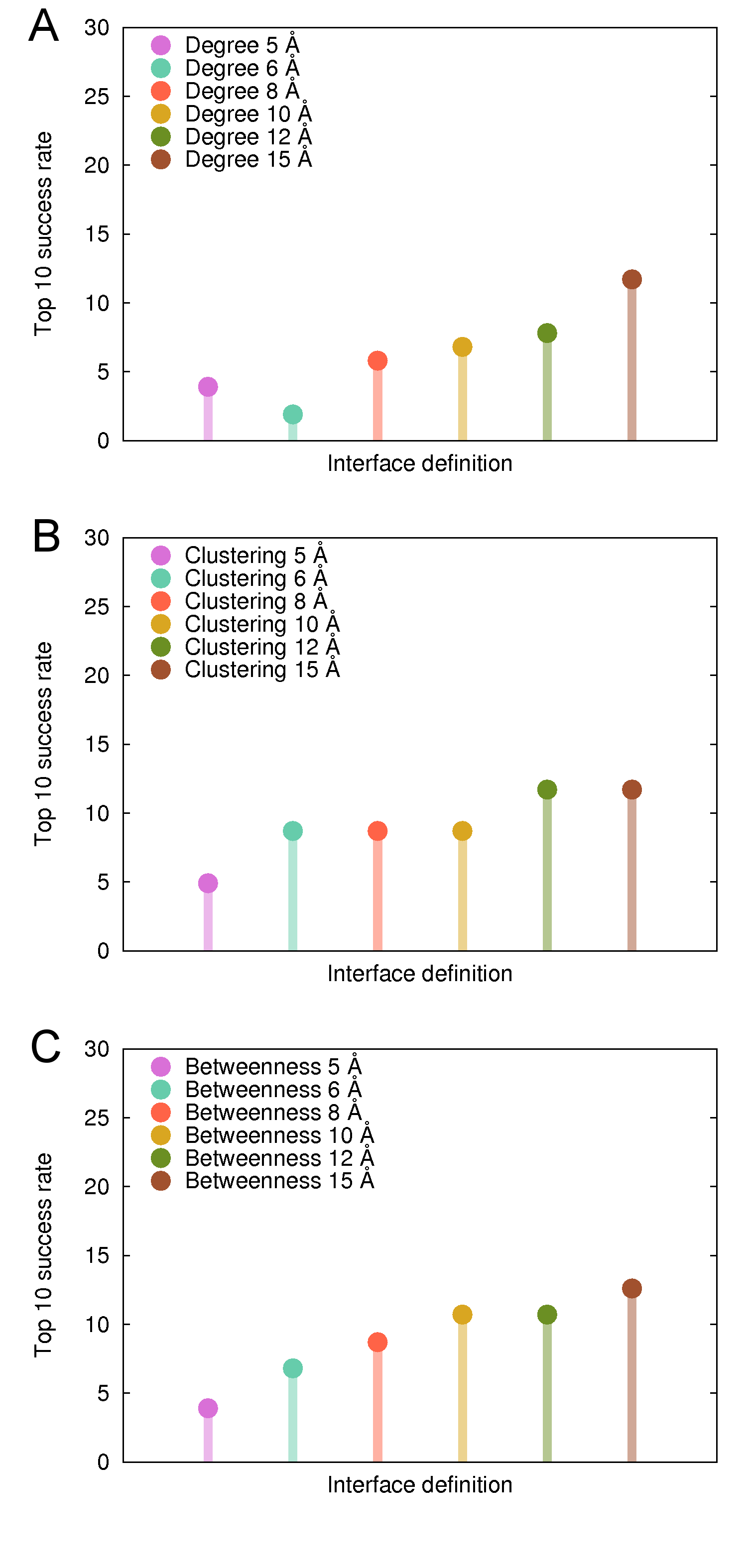


**Figure S6.- Performance of network-based scoring methods**

Top 10 success rates when scoring the docking poses using different contact distances to define the docking interface residues with the (A) degree values. (B) clustering values, (C) betweenness values


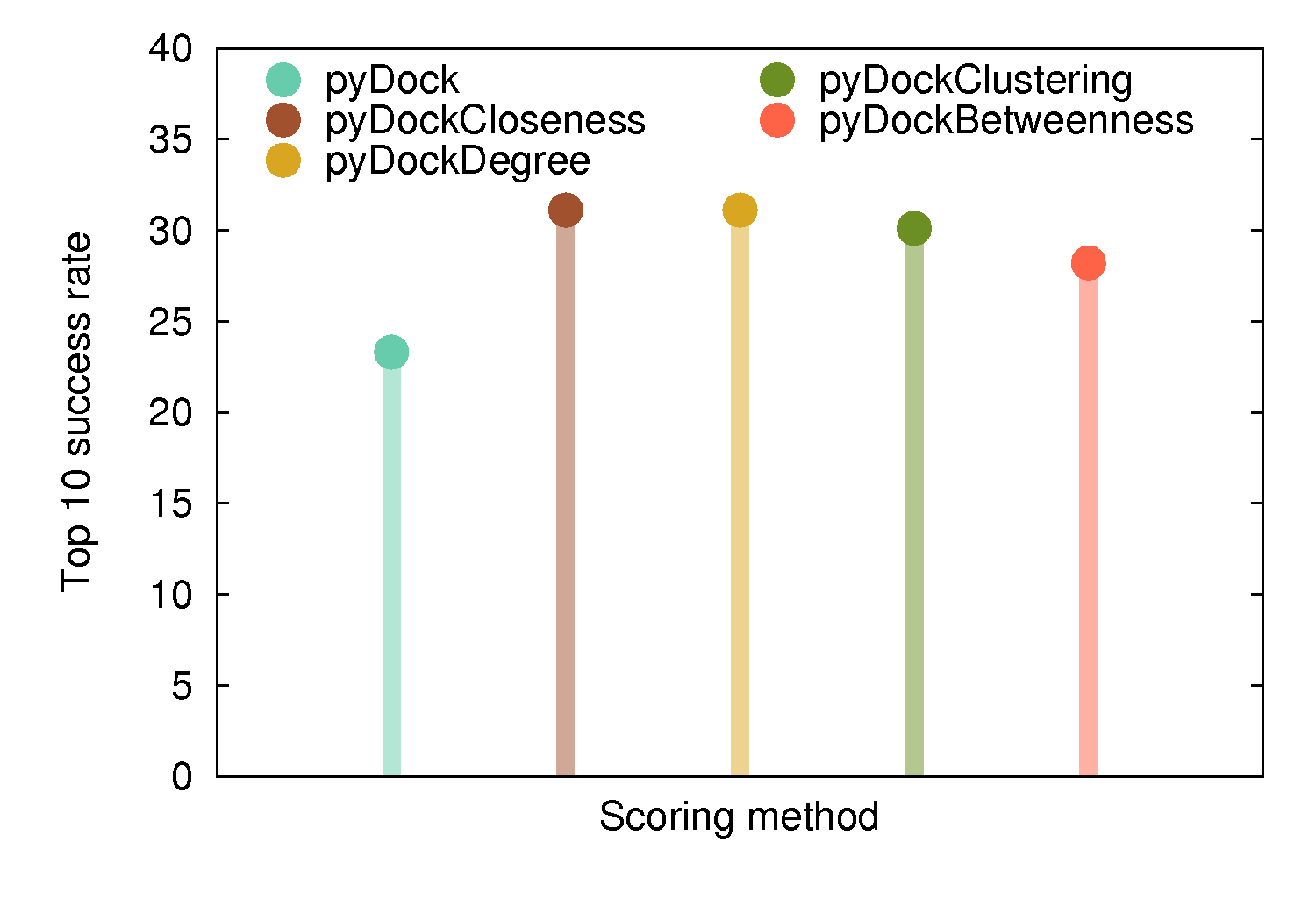


**Figure S7.- Scoring performance of the combination of network-based methods and pyDock**

Top 10 success rates of pyDockCloseness, pyDockDegree, pyDockClustering, pyDockBetweenness and pyDock


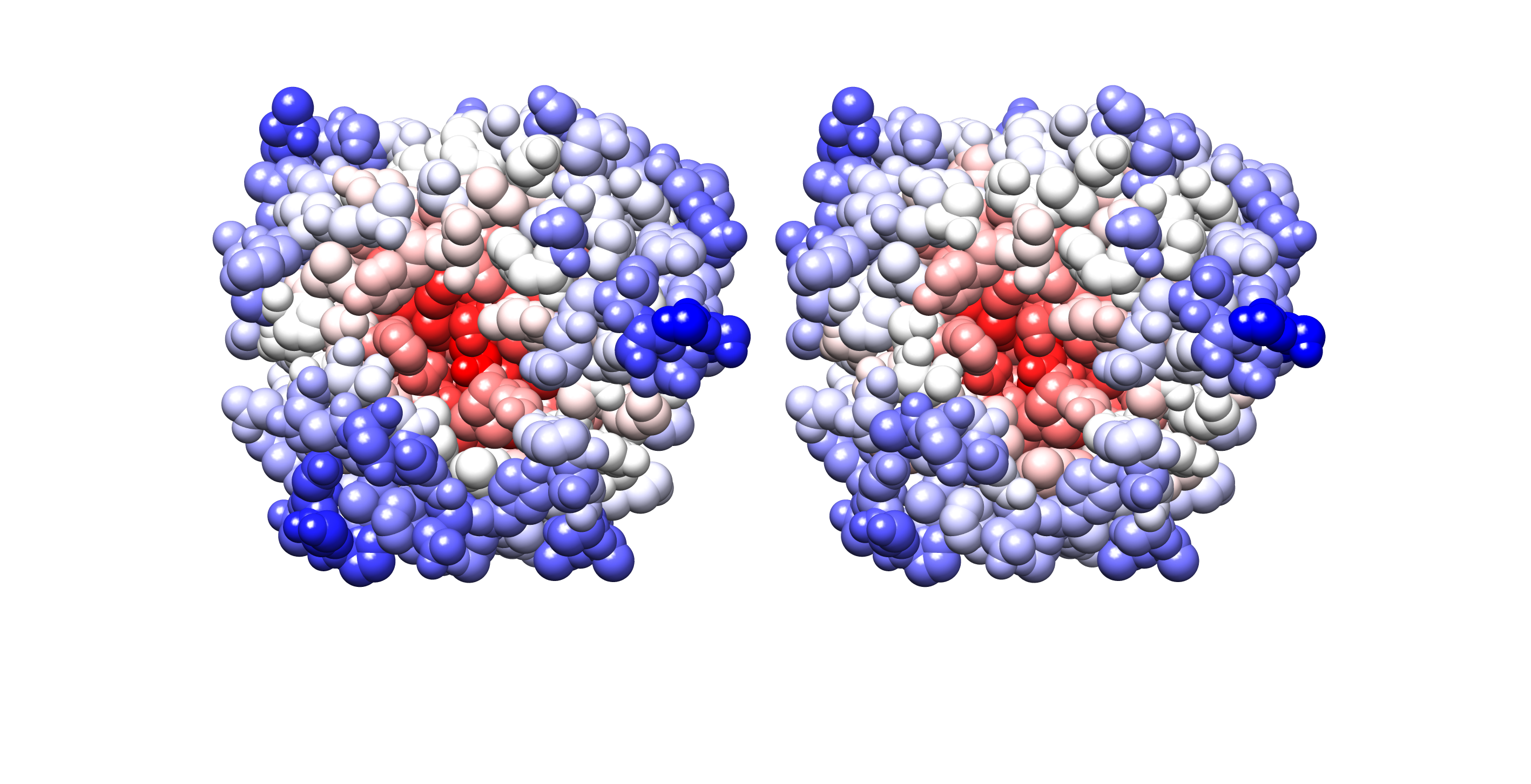


**Figure S8.- Comparison of Cα and Cβ based topology networks**

Topology networks for unbound Subtilisin (complex PDB code 2SIC) with nodes defined based on Cα atoms (left panel) or Cβ atoms (right panel). Residues are colored according to the *closeness* value of their corresponding node, from high (in red) to low (in blue) values.
